# Complete 4.55-Megabase-Pair Genome of “*Candidatus* Fluviicola riflensis,” Curated from Short-Read Metagenomic Sequences

**DOI:** 10.1128/genomeA.01299-17

**Published:** 2017-11-22

**Authors:** Jillian F. Banfield, Karthik Anantharaman, Kenneth H. Williams, Brian C. Thomas

**Affiliations:** a University of California, Berkeley, California, USA; b Lawrence Berkeley National Laboratory, Berkeley, California, USA; c University of Melbourne, Melbourne, Victoria, Australia

## Abstract

We report the 4.55-Mbp genome of “*Candidatus* Fluviicola riflensis” (*Bacteroidetes*) that was manually curated to completion from Illumina data. “*Ca*. Fluviicola riflensis” is a facultative anaerobe. Its ability to grow over a range of O_2_ levels may favor its proliferation in an aquifer adjacent to the Colorado River in the United States.

## GENOME ANNOUNCEMENT

We used genome-resolved metagenomics to investigate a bacterium that is abundant in an aquifer near Rifle, CO, USA. Previously, ~1,300 unique draft and 6 complete genomes were reported from the groundwater and sediments of this site, making it one of the most comprehensively genomically described environments to date (1–5). Groundwater was collected during an episode in which dissolved O_2_ concentrations were seasonally elevated for a short duration. Cells were captured onto 1.2-, 0.2-, and 0.1-µm filters. Using IDBA-UD, 150-bp paired-read Illumina data were assembled (3).

The initial genomic bin comprised six scaffolds of 107 to 1.7 Mbp (3). Local assembly errors were investigated using ra2 (https://github.com/christophertbrown/fix_assembly_errors) and unresolved problems corrected by manual analysis. A large repeat-bearing gene was deciphered based on paired-read information, and two rRNA operons were recovered by sequential sequence extension. All scaffolds were joined based on sequence overlap with paired-read support. The rRNA genes shared highest similarity with those of *Fluviicola taffensis* DSM 16823, for which there is a complete genome (96% 16S rRNA identity [6]). The complete (circularized, no gaps) genome is 4.551 Mbp (42.82% GC content), has 36 tRNA genes, and has 3,910 predicted proteins. To our knowledge, complete curation of such a large genome from metagenomic short-read data is unprecedented. Genome accuracy was confirmed based on the cumulative GC skew from the origin to the terminus of replication. The proposed species name, “*Candidatus* Fluviicola riflensis,” honors the history of microbiology at the Rifle site.

We used iRep and bPTR to evaluate the *in situ* replication rate (7). Both values were ~1.1, indicating that a minority of cells were actively growing. Given its already high abundance, this may reflect substrate limitation.

We predict that “*Ca*. Fluviicola riflensis” can make nucleic acids and most amino acids and produce a Gram-negative cell envelope containing phosphatidyl ethanolamine, phosphatidylcholine, and lycopene (possibly with O-antigen 2,3-diacetamido-2,3-dideoxy-α-d-mannuronate). It has genes for pili and gliding motility, 41 PKD domain proteins, and many other large putative cell surface proteins, including a 2,692-amino acid FG GAP domain-containing repeat protein probably involved in adhesion.

Unlike *F. taffensis*, we infer that “*Ca*. Fluviicola riflensis” is a facultative anaerobe. Encoded is the potential for breakdown of cellulose, cellobiose, hemicellulose, and chitobiose; the glycolysis pathway; complete tricarboxylic acid (TCA) cycle; and an aerobic electron transport chain. The genome encodes enzymes for radical detoxification, including alkyl hydroperoxide reductase, superoxide dismutase, and multiple cytochrome *c* peroxidases. It appears to have genes for conversion of nitroalkanes to nitrite, the degradation of aromatic compounds, and two operons involved in the degradation of phenylacetate via an oxygenase (aerobic pathway [8]). Consistent with the ability to grow without O_2_, it synthesizes menaquinone (normally involved in an anaerobic electron transport chain [9]) and has the capacity for fermentation to acetate and d-lactate. Also present are genes involved in amino acid degradation, which could represent a primary energy source.

### Accession number(s).

The genome of “*Candidatus* Fluviicola riflensis” is available at the DDBJ/EMBL/GenBank database under accession number CP022585 (version CP022585.1) and at ggKbase.
